# Role of laparoscopy in evaluation of chronic pelvic pain

**DOI:** 10.4103/0972-9941.18995

**Published:** 2005-09

**Authors:** Shripad Hebbar, Chander Chawla

**Affiliations:** Department of O & G, Kasturba Medical College, Manipal; 1 Department of O & G, Manipal Teaching Hospital, Pokhara

**Keywords:** chronic pelvic pain, endometriosis, laparoscopy, pelvic adhesions

## Abstract

**Introduction:**

Chronic pelvic pain (CPP) is a common medical problem affecting women. Too often the physical signs are not specific. This study aims at determining the accuracy of diagnostic laparoscopy over clinical pelvic examination.

**Settings and Design:**

A retrospective study of patients who underwent diagnostic laparoscopy for CPP.

**Materials and Methods:**

The medical records of 86 women who underwent laparoscopic evaluation for CPP of at least 6-month duration were reviewed for presentation of symptoms, pelvic examination findings at the admission, operative findings and follow up when available.

**Statistical analysis used:**

McNemar Chi-square test for frequencies in a 2 × 2 table.

**Results:**

The most common presentation was acyclic lower abdominal pain (79.1%), followed by congestive dysmenorrhoea (26.7%). 61.6% of women did not reveal any significant signs on pelvic examination. Pelvic tenderness was elicited in 27.9%. Diagnostic laparoscopy revealed significant pelvic pathology in 58% of those who essentially had normal pervaginal findings. The most common pelvic pathology by laparoscopy was pelvic adhesions (20.9%), followed by pelvic congestion (18.6%). Laparoscopic adhesiolyis achieved pain relief only in one-third of the women.

**Conclusion:**

The study revealed very low incidence of endometriosis (4.7%). Overall clinical examination could detect abnormality in only 38% of women, where as laparoscopy could detect significant pathology in 66% of women with CPP. This shows superiority of diagnostic laparoscopy over clinical examination in detection of aetiology in women with CPP (P < 0.001). Adhesiolysis helps only small proportion of women in achieving pain control.

Chronic pelvic pain (CPP) is one of the commonest symptomatology in gynaecological out patient clinics. It accounts for 10% of office visits to gynaecologists[1] and general clinics.[2] According to Renaer,[3] CPP accounts for about a quarter of out patient consultations in general gynaecological practice. Arbitrarily CPP is defined as 6 months or more of constant or intermittent, cyclic or acyclic pelvic pain that includes dysmenorrhoea, deep dypareunia and intermenstrual pain.[4,5] However, the objective evaluation of pain poses a complex task as most of the times physical signs are absent. Most of the times patients are treated symptomatically or referred to psychiatrist as somatoform disorder without adequate diagnostic evaluation.[6]

Laparoscopy is a valuable tool in the evaluation of undiagnosed CPP. It can establish a definitive diagnosis and modify the treatment without resorting to exploratory laparotomy. It is also an extremely valuable adjunct in gynaecologist's armamentarium especially in confirming minimal disease and adhesions, which cannot be revealed sonographically. The following study is an attempt in understanding the aetiology of such a complex and perplexing problem in day-to-day gynaecological practice.

## MATERIALS AND METHODS

Between January 1999 and December 2003, 86 women were admitted to Department of OBG, Manipal Teaching Hospital, Pokhara for laparoscopic evaluation of CPP of at least 6-month duration. Their medical records were reviewed for presentation of symptoms, pelvic examination findings at the admission, operative findings and follow up when available. [Table 1] shows presenting symptoms at the time of admission.

**Table 1:** Main symptoms in 86 women with CPP

Symptoms	*n*	%
Acyclic lower abdominal pain	68	79.1
Congestive dysmenorrhoea	23	26.7
Deep dyspareunia	9	10.5
Spasmodic dysmenorrhoea	4	4.7

* 18 patients (20.9%) had more than one symptoms.

The most common presentation was lower abdominal pain (79.1%) bearing no relation to the menstrual cycles. Four women with severe spasmodic dysmenorrhoea were admitted for further evaluation, as they did not respond to NSAIDs and combination pills. Psychosomatic disorders were ruled out by referring to the psychiatrist. Fair antibiotic trial was given for adequate duration in those who had questionable evidence of PID and were considered for laparoscopy only after they failed to respond for medical therapy to redefine the diagnosis. Patients with superficial dyspareunia were not included in the study. Surgical referral was done whenever pain was predominant in one of the iliac fossae and with previous history of appendicectomy.

Age ranged from 19 to 48 years, with mean of 28 years. Parity ranged from 0 to 8 with mean of 2.8. Nine women were nulliparae. Nineteen had history of previous surgery (tubal sterilization-11, caesarean section-5, appendicectomy-2 and ovarian cystectomy-1). Nine women had history of first trimester MTP. Eight had undergone dilatation and curettage for menstrual irregularities.

[Table 2] shows the main presenting symptoms in these 86 women. Pelvic tenderness on pervaginal examination was the most common finding (27.9%), followed by forniceal fullness (15.1%). Fifty-three (61.6%) women did not reveal any significant signs.

**Table 2:** Pervaginal findings in 86 women with CPP

Signs	*n*	%
Pelvic tenderness		
Localized to one fornix	7	8.1
Bilateral	9	10.5
Diffuse	8	9.3
Cul-de-sac nodularity	3	3.5
Fixed retroverted uterus	2	2.3
Forniceal fullness		
- Unilateral	8	9.3
- Bilateral	5	5.8
No significant findings	53	61.6

* 9 (10.5%) had more than one sign.

Diagnostic laparoscopy was performed under general anaesthesia. A 5-mm Karl Stortz 30° angle double port laparoscope was used. Carbon dioxide pneumoperitoneum was accomplished with a 15-gauge Verres needle. When manipulation of the pelvic organs was required for improved visualization, a second puncture site was established lateral to left rectus muscle under vision taking care to avoid injury to inferior epigastric artery. A third port was established similarly on right side whenever an operative procedure was undertaken such as, fulguration, adhesiolysis and cyst wall puncture. Under surface of liver and diaphragm was always inspected for adhesions before completing procedure.

## RESULTS

The main objective of this study was to correlate laparoscopic findings with preoperative pelvic findings, to determine the type of pathology existing and to re-evaluate the treatment strategy. Of 86 women enrolled for study, only 33 (38%) had significant findings on preoperative pelvic examination. In contrast 57 (66%) had abnormal findings on laparoscopy. Conversely 53 (62%) had normal preoperative pelvic findings and 29 (33%) were negative for pathology on laparoscopy.

[Table 3] shows correlation between pelvic and laparoscopy examination findings. Fifty-eight per cent (31/53) of those who had normal preoperative pelvic findings and 79% (26/33) of those with abnormal preoperative pelvic findings had significant pelvic pathology on laparoscopy. The error in pelvic examination in symptomatic patients varied from 21% (normal findings) to 58% (abnormal findings).

**Table 3:** Correlation between pelvic examination and laparoscopic findings

	Laparoscopic findings			

	Normal (29)		Abnormal (57)	
Preoperative PV examination	No.	%	No.	%
Normal (*n* = 53)	22	42	31	58
Abnormal (*n* = 33)	7	21	26	79

[Table 4] shows correlation between laparoscopic and pelvic examination findings. Preoperative examination was abnormal in 24% (7/29) in those who had no pathology on laparoscopy. Conversely out of 57 women who had abnormal findings on laparoscopy 54% (31/57) had essentially no findings on pervaginal examination.

**Table 4:** Correlation between laparoscopic and pelvic examination findings

	Preoperative PV examination			

Laparoscopic findings	Normal (53)		Abnormal (33)	
	No.	%	No.	%
Normal (n = 29)	22	76	7	24
Abnormal (n = 57)	31	54	26	46

χ^2^ = 86, *P* < 0.001 (McNemar Chi-square test).

To summarize, clinical examination could detect abnormality only in 33 (38%) women, where as laparoscopy could detect pathology in 57 (66%) women with CPP. This shows superiority of diagnostic laparoscopy over clinical examination in detection of aetiology in these women, which is statistically agreeable (χ^2^ = 86, *P* < 0.001, McNemar Chi-square test).

Actual laparoscopic findings are shown in [Table 5]. The most common pelvic pathology seen in this study was pelvic adhesions (20.9%) followed by pelvic congestion (18.6%). The diagnosis of PID was considered if one of the following criteria were present; hyperaemic, oedematous and congested fallopian tube, pus oozing from fimbriae and presence of hydro/pyosalpinx. Pelvic congestion was diagnosed in the presence of bulky, boggy uterus, broad ligament and infundibulopelvic ligament varicosities. However none of the patients had undergone pelvic venography for diagnosis of pelvic congestion before the laparoscopy procedure as this procedure was not yet available in the institution where the study was carried out and the diagnosis was based on the laparoscopist's expertise.

**Table 5:** Laparoscopic findings in 86 women with CPP

			Pelvic examination	

Lap findings	*n*	%	Normal	Abnormal
Pelvic adhesions	18	20.9	10(56%)	8(44%)
Pelvic congestion	16	18.6	9(56%)	7(44%)
PID	8	9.3	3(38%)	5(62%)
Ovarian pathology	7	8.1	4(57%)	3(43%)
Endometriosis	4	4.7	2(50%)	2(50%)
Uterine fibroid	3	3.5	2(67%)	1(33%)
Genital tuberculosis	1	1.2	1(100%)	–
Normal findings	29	33.7	22(76%)	7(24%)
Total	86		53(62%)	33(38%)

Seven patients had ovarian pathology (simple follicular cyst 4, polycystic ovaries 2, ruptured corpus luteal cyst 1), though theoretically they could not explain origin of pelvic pain. The maximum size of the cyst was 4 cm and all cysts were aspirated. When the cases were followed up subsequently, none had recurrence of the cyst. Surprisingly the incidence of endometriosis in this study was only 4.7%. They presented as jelly like deposits, powder black burns, white-scarred areas and puckered lesions. Only two of them had positive findings preoperatively in the form of cul-de sac nodularity and all belonged to stage-I disease by revised American fertility society (AFS) classification. Three patients had fibroid uterus, not diagnosed initially even by sonography. Only one had doubtful cul-de-sac nodularity in preoperative pelvic examination. All three myomas measured less than 2 cm and obviously were subserous.

[Table 6] shows the type of previous operations in patients with adhesions. In 38.9% no obvious cause could be detected. This may be attributed to ‘silent PID’ resulting from Chlamydia and Mycoplasma group of organisms. Tubal ligations (none were laparoscopic sterilizations) accounted for 22.2% of cases. None of the adhesions were associated with bowel obstruction.

**Table 6:** Nature of previous ‘surgery’ in patients with adhesions (*n* = 18)

	Grade I	Grade II	Grade III	Total	Overall %
Tubal ligations	4	0	0	4	22.2
Caesarean section	1	1	1	3	16.7
Dilatation and curettage	2	0	0	2	11.1
Appendicectomy	0	1	0	1	5.6
Ovarian cystectomy	0	1	0	1	5.6
None	4	3	0	7	38.9
Total	11	6	1	18	100

Adhesions were classified according to adhesion scoring method of AFS,[7] i.e. grade I (localized covering one-third of adnexa), grade II (moderate, covering one-third to two-thirds of the adnexa) and grade III (extensive adhesions covering more than two-thirds of adnexa). All cases of grade-I adhesions were lysed at the time of diagnostic laparoscopy. There were three cases of grade-II adhesions and we could not release adhesions completely in two cases mainly because of close proximity to the rectum fearing bowel injury. Grade-III adhesion was present only in one case who had undergone caesarean section previously and adhesiolyis was not attempted as whole pelvis was obscured. We have tried a course of steroids for adhesiolysis failure, but results are not promising. At the time of reporting this paper, only 33% (4 out of 12) of those who underwent adhesiolyis seem to be benefited from the procedure at the end of 1 year of observation.

## DISCUSSION

This study confirms the previous observations that laparoscopy is an effective tool in the evaluation of women with CPP.[8,9] The error in diagnosis at preoperative pelvic examination in this study ranged from 21 to 58%. There was better correlation between abnormal preoperative pelvic examination and abnormal laparoscopic findings (79%, [Table 3]. Similar experiences were reported by other authors.[10,11] [Table 7] shows observations made regarding negative laparoscopy in various studies.

**Table 7:** Pathology identified during laparoscopy

		No. of Patients	Patients with negative	Patients with organic lesions

Study	Year		finding (%)	(%)
Kleinhaus et al.[12]	1977	50	44	56
Goldstein et al.[9]*	1980	140	14	86
Chatman and Ward[13]*	1982	73	12	88
Vercellini et al.[14]	1989	47	40	60
Porpora and Gomel[15]	1997	1336	36	64
Present study	2005	86	34	66

* Series of adolescent patients.

An interesting observation made during this study was that the incidence of endometriosis is very low in this part of Nepal (4.7%). This may be due to prevalence of early child bearing, prolonged breast feeding[16] and higher use of Depo-Provera for contraception in Nepalese women. Family Planning Association Statistics for the year 2003 showed that 48% of women preferred Depo-Provera as the method of choice for contraception.[17] Since FDA approval for contraceptive use in 1992, Depot Medroxy-Progesterone Acetate (DMPA or Depo-Provera) has been used by millions of women worldwide and its long term benefit in reducing the incidence of dysmenorrhoea, menorrhagia, endometriosis, endometrial hyperplasia, ovulatory pain and pain associated with ovarian adhesive disease is well known.[18] The incidence of endometriosis by different authors is shown in [Table 8].

**Table 8:** Incidence of endometriosis in series of patients who underwent laparoscopy for CPP

Author	Year	No. of patients	% with endometriosis
Goldstein et al.[9]	1980	140	47
Chatman and Ward[13]	1982	43	65
Kresh et al.[4]	1984	100	32
Rapkin[5]	1986	100	37
Rajan[19]	1988	631	29
Vercellini et al.[14]	1989	47	38
Porpora and Gomel[15]	1997	1336	31
Kontoravdis et al.[11]	1999	98	25
Present study	2005	86	4.7

## CONCLUSION

One of the most perplexing problems facing the gynaecologist is the patient who has CPP. When there are objective physical signs and symptoms, the accuracy for diagnosis of origin of pain is increased. However, too often the physical signs are not specific; e.g. pelvic tenderness, pelvic congestion,[20] questionable pelvic mass and adnexal fullness. The present study indicates that laparoscopy is an excellent tool in evaluation of patients with pelvic pain, because diagnosis and often treatment (e.g. adhesiolysis,[21] cyst aspiration) can be accomplished in one sitting, without subjecting the patients to exploratory laparotomy. Endometriosis can be diagnosed only by laparoscopy, and it can often be treated at the time of diagnosis by either electrocoagulation or laser vaporization. In fact there is some suggestion in the literature that entity of CPP is best investigated laparoscopically before any treatment is planned.[22] Recently laparoscopic pain mapping[23,24] under local anaesthesia and sedation appears to be promising to improve the accuracy of laparoscopy as a diagnostic tool in CPP.

At present the role of adhesiolysis in treatment of CPP is still controversial. It is not shown to be effective in achieving pain control in randomized clinical studies.[25] Second look laparoscopy studies reveal a surprising amount of adhesion reformation despite good surgical technique.[26,27] In the present study, only 33% had pain relief at the end of 1 year of observation, however the number studied is too small and a larger prospective study may be needed to derive statistically significant conclusion.
